# A flow feature detection method for modeling pressure distribution around a cylinder in non-uniform flows by using a convolutional neural network

**DOI:** 10.1038/s41598-020-61450-z

**Published:** 2020-03-10

**Authors:** Shuran Ye, Zhen Zhang, Xudong Song, Yiwei Wang, Yaosong Chen, Chenguang Huang

**Affiliations:** 1 Key Laboratory for Mechanics in Fluid Solid Coupling Systems, Institute of Mechanics, Chinese Academy of Sciences, Beijing, 100190 China; 2 School of Engineering Science, University of Chinese Academy of Sciences, Beijing, 100049 China; 3 College of Engineering, Peking University, Beijing, 100871 China

**Keywords:** Fluid dynamics, Mechanical engineering

## Abstract

In a myriad of engineering situations, we often hope to establish a model which can acquire load conditions around structures through flow features detection. A data-driven method is developed to predict the pressure on a cylinder from velocity distributions in its wake flow. The proposed deep learning neural network is constituted with convolutional layers and fully–connected layers: The convolutional layers can process the velocity information by features extraction, which are gathered by the fully-connected layers to obtain the pressure coefficients. By comparing the output data of the typical network with Computational Fluid Dynamics (CFD) results as reference values, it suggests that the present convolutional neural network (CNN) is able to predict the pressure coefficient in the vicinity of the trained Reynolds numbers with various inlet flow profiles and achieves a high overall precision. Moreover, a transfer learning approach is adopted to preserve the feature detection ability by keeping the parameters in the convolutional layers unchanged while shifting parameters in the fully-connected layers. Further results show that this transfer learning network has nearly the same precision while significantly lower cost. The active prospects of convolutional neural network in fluid mechanics have also been demonstrated, which can inspire more kinds of loads prediction in the future.

## Introduction

In the research field of fluid mechanics, the loads modeling of flow field has always been an important issue in complicated flow field. Fortunately, the emerging deep learning methods could provide more ideas for it. The big data era and the significantly improved computing power have laid a good foundation for the machine learning as well as its related applications. Therefore, the provided massive labeled flow field data makes it promising to expand the machine learning method to fluid mechanics. Early attempts were made mainly focusing on turbulence modeling. Tracey *et al*.^1^ used neural networks with a single hidden layer to simulate terms in Spalart-Allmaras Reynolds Averaged Navier-Stokes (RANS) model, which suggests the possibility of neural networks in turbulence modeling. Ling *et al*.^2^ proposed a multiplicative layer embedded with Galilean invariance to predict the Reynolds stress anisotropy tensor. This neural network architecture provides improved accuracy in Reynolds stress modeling than their previous work using random forest. Wang *et al*.^3^ reconstructed the discrepancies in the RANS-simulated Reynolds stress using high-fidelity Direct Numerical Simulation (DNS) data based on machine learning methods. This method offered a different way to interpret the improved prediction.

Above all, more study needs to be done to figure out the possibilities machine learning owns in fluid mechanics, not only on turbulence modeling, but on flow feature study as well. Success on ImageNet Classification with deep convolutional neural networks makes the convolutional neural network popular for its fewer connections and parameters^4^. Umetani and Bickel^5^ proposed a data driven method to predict the fluid flow around three-dimensional objects. A novel PolyCube maps-based parametrization was adopted to instantly calculate the nonlinear response of the flow using a Gaussian process regression. Ströfer *et al*.^6^ used convolutional neural network to identify the features in fluid flow. This attempt received good results, which provides a general method to flow feature identification study even for distinguishing between similar ones. Jin *et al*.^7^ also used the convolutional network to predict the velocity field around the cylinder. This method can detect the invariant features of the small translations in the temporal dimension of pressure fluctuations on the cylinder. Liu *et al*.^8^ proposed a shock wave detection method based on CNN, which has better performance on detection result than the first step to introduce deep learning into shock detection method with less time consumption than the traditional approach. These papers proved that the convolutional neural network have the critical advantages on feature detection in fluid mechanics.

Therefore, we began to focus on the relationship between the flow structure and the concerned flow statistics. The convolutional neural network is constructed in hope of finding the mapping relation between them. Flow around a cylinder is taken as the example to discuss the proposal. This type of flow is a common flow structure owning certain complexity as well. Based on the classic non-uniform flow field, the network between the flow structure and the flow concerned amount is constructed. This CNN-based deep learning method links the flow field to the pressure on cylinder by an intermediate variable which presents the feature learned from the flow field.

The rest of this paper is described as follows: In Sect.2, we describe flow data and the configuration of the CNN. The results of the network and the evaluation are presented in Sect.3, and the explanation of the convolution operation is discussed in Sect.4. In Sect.5, transfer learning is introduced to improve the network efficiency. Finally, Sect.6 concludes the paper.

## Methodology

### Flow modeling & data acquisition

The flow around a cylinder has always been a typical project in fluid mechanics, due to its critical influence in engineering problems. When the Reynolds number increases, the wake flow passing the cylinder turns into an unsymmetrical state, generally presented as vortex shedding alternately, which will cause the fluctuating pressures on the surface of the cylinder^9^.

**Figure 1:**
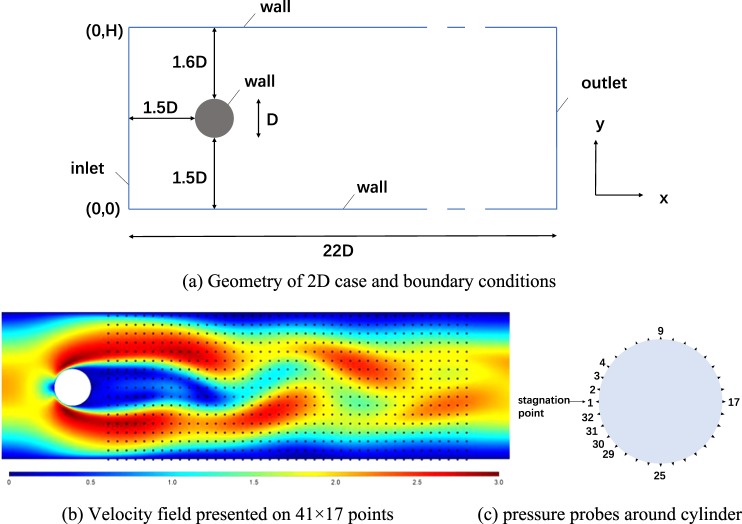
Flow geometry and probes distribution in the flow around a cylinder. (**a**) Geometry of 2D case and boundary conditions. (**b**) Velocity field presented on 41 × 17 points in the wake flow of the cylinder. (**c**) Indexes and locations of pressure probes around cylinder.

Therefore, we focus on the flow around a circular cylinder whose pressure on the surface changes along with the velocity field. For the flow around a cylinder with a circular cross-section, the two-dimensional case is adequate to express flow features^10^. The flow geometry along with boundary conditions are shown in Fig. 1a. The profile of inlet horizontal velocity is set as a parabolic function: $u(y)=\frac{4{u}_{m}\,y(H-y)}{{H}^{2}}$. And the vertical component of velocity at inlet is set as zero.

Reynolds number ranges from 1000 to 10000, which is defined by ${\rm{Re}}=\bar{u}D/\upsilon$ with the mean velocity $\bar{u}=2u(H/2)/3$ and cylinder diameter *D* = 0.1. For each Reynolds number, flow with 300 frames are calculated where time step dt = 0.005 s. Pressure coefficient *C*_*p*_ is chosen as the network target considering its non-dimensional property, and is defined as ${C}_{p}=\frac{P}{\frac{1}{2}\times \rho \times {\bar{u}}^{2}}$, where P is the local pressure and *ρ* is the fluid density. To match the deep learning configuration, data we use for the network should be preprocessed. The wake flow area in range 0.3 ≤ x ≤ 1.3 and 0 ≤ y ≤ 0.4 is chosen as the velocity field area. A mesh of 41 × 17 point probes is created using interpolation method at the distance of 0.025 to represent the velocity field (Fig. 1b), and 32 probes are located on the cylinder surface to monitor the pressure coefficient on the cylinder, respectively (Fig. 1c).

**Figure 2:**
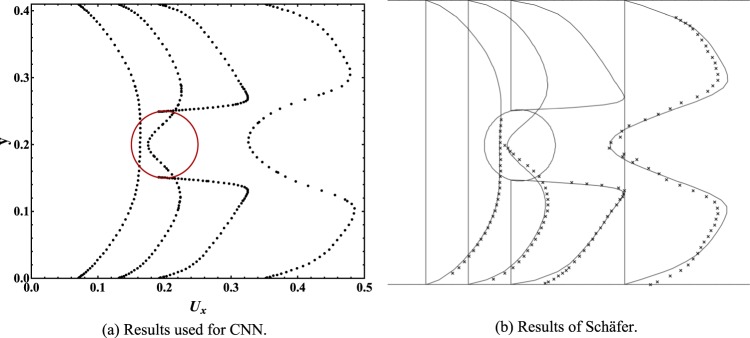
Velocity profile of flow around a cylinder. (**a**) Numerical simulation results used for CNN. (**b**) Numerical simulation and experimental results of Schäfer.

The result of our numerical simulation in the flow around the cylinder is shown in Fig. 2 to discuss its validity. Figure 2a is the velocity profile in our numerical simulation, while Fig. 2b is the velocity profile carried out by Schäfer^10^. By comparing the velocity profile results around the cylinder, we find that our result shows good consistency with the Schäfer’s data.

### Construction of network

**Figure 3:**
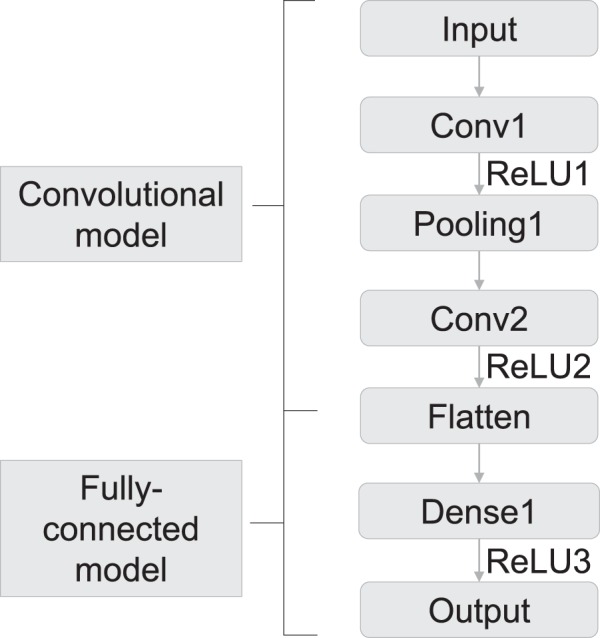
The architecture of the CNN to train the model.

**Figure 4:**
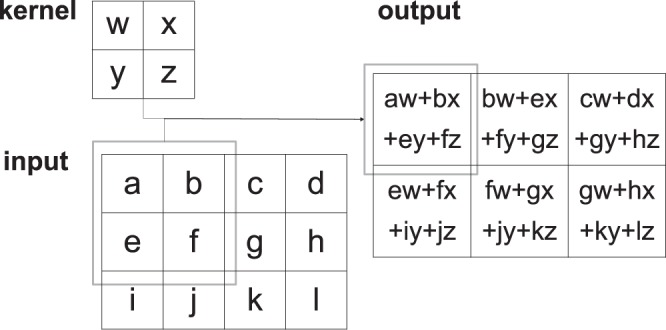
Convolution: the kernel size is 2 × 2.

The architecture of the CNN is shown in Fig. 3. “Conv” denotes convolutional layer; “ReLU” denotes rectified linear unit; “Pooling” denotes max pooling layer; “Dense” denotes fully connected layer^11^. The network is composed of two main parts. The convolutional layers are an important part in a CNN. Convolution is a mathematical operation, which presented as a weighted average method (Fig. 4). By allocating the weight function as *w*, its common expression is presented as $s(t)=(r\ast g)(t)$. In this expression, the function *r* in the convolution is often referred as the input, while the function *g* is referred as the kernel. Considering the discrete time term as well as the two-dimensional kernel for the convolutional operation, this expression can be written as: $s[i,j]=(I\ast K)(i,j)={\sum }_{m}{\sum }_{n}I[m,n]K(i-m,j-n)$^4^. Since the kernel size is far smaller than the input size, it leads to the critical improvements: sparse interactions and parameter sharing. Another improvement in efficiency called equivariant representations is achieved by pooling layer. Pooling operation makes the output of the network replaced by the overall statistical characteristics of adjacent outputs. Max pooling that gives the max value of the given location is one of the pooling operations^12^. In the convolutional model shown in Fig. 3, the CNN architecture is constructed considering both the classical simple network LeNet5 and the input size of our network, while the maxpooling whose pooling size is 2 × 2 and stride is 1 is adopted.

Also, the fully-connected layers are a basic network form in deep learning methods, which is also known as the multilayer perceptron. Each node in one layer is connected to every node in the following layer. This type of network is inspired by the neuron model in biological neural networks, whose function is to pass information through connections. As for the neurons in neural network model, the relation is expressed as $f(a;w;b)={a}^{T}w+b$, where *w* is the weight and *b* is the bias. In the network described in Fig. 3, the dense layer with 128 neurons is chosen.

In order to allow the neuron performed as nonlinear relation, activation function is added following both the convolutional layer and the fully-connected layer. The activation function in the network is Rectified Linear Unit (ReLU) whose expression is: $f(z)=max(0,z)$. The results of the convolutional layer are collected by the fully-connected layer to get the final output. Hence the whole structure of the CNN is constructed.

### Training configurations

Considering the construction of the neural network under the purpose of pressure coefficient prediction, the input of this network is supposed to be the velocity of the flow field. The velocity is presented as a three-dimensional matrix [x, y, **U**], where **U** stands for {*u*, *v*} in the two-dimensional flow. It has been noted that all the inputs and outputs are non-dimensional. Therefore, all data needs to be preprocessed, non-dimensionalized by ${\rm{\rho }},\,\bar{u}$ and *D*.

The loss function is a non-negative real-valued function which measures the deviation between the predicted value and the CFD results^13^. The loss function is chosen as the root mean square error (RMSE). It presented as $RMSE=\sqrt{\mathop{\sum }\limits_{i=1}^{n}{({y}_{i}-{\tilde{y}}_{i})}^{2}\cdot \frac{1}{n}}$, where *y*_*i*_ is the CFD results and ${\tilde{y}}_{i}$ is the output of the network^14^. Thus this training process is converted into an unconstrained optimization problem, finding the proper variables which lead to the minimum value of the loss function. Gradient descent algorithms are basic methods to attain the optimized value and Adam algorithm is one of the most universal methods for its effectiveness. It is an algorithm for first-order gradient-based optimization of stochastic objective functions based on adaptive estimates of lower-order moments. The learning rate of the Adam algorithm is 0.001 and the beta value is beta1 = 0.9, beta2 = 0.999. Monitoring the continually decreasing RMSE, the neural network gradually approaches to the real mapping relation^15^. Noted that the mini-batch training is used whose batch size used for training is 64. The CNN training process is carried out with TensorFlow, an end-to-end open source platform for machine learning^16^.

## Result of Pressure Modeling with The Typical CNN Network

### Result on basic data

The training set is the flow around cylinder cases at Reynolds number equaling to 1000, 3000, 5000, 7000, and 9000, while the testing set is the flow around cylinder at Reynolds number equals to 2000, 4000, 6000, 8000, and 10000. The data is acquired under the condition described in Section 2.1. The same cylinder was used while the different Reynolds number is achieved with different viscosity coefficient. Noted that the Re = 10000 is an extrapolate testing data set which can better illustrate the feasibility range of this method. For each Reynolds number, 300 frames are calculated where dt = 0.005 s. Therefore, the size of training samples and the testing samples are both 1500.

**Figure 5:**
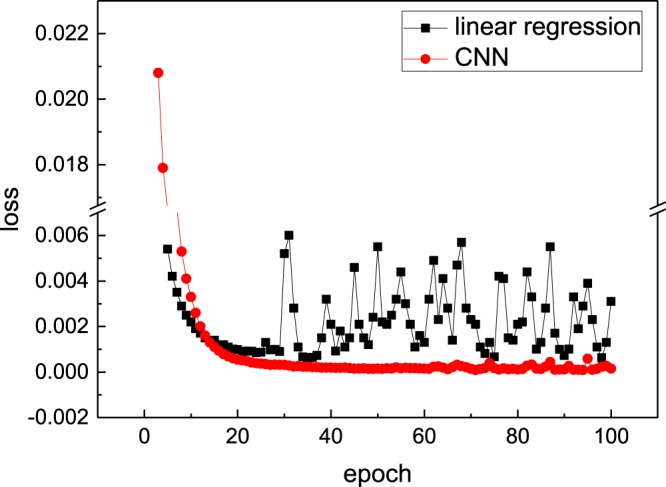
Convergence of the CNN and the linear regression model.

With the iteration of the losses and the weights in the network, this network gets closer to the real mapping relation. By back propagation to the weights in each layer, the loss function which stands for the RMSE drops rapidly. As shown in Fig. 5, the training error for entire dataset decreases with the increasing number of training epoch. When the epoch reaches to 50, the loss of this network estimates to 2 × 10^−4^. It means the root mean square error between the real pressure coefficient and the predicted pressure coefficient is 2 × 10^−4^. To better understand the CNN model which is not only a physics informed neural network, but has improved performance as well, a comparison with linear regression is carried out. The linear regression is constructed by the dense layer in the network without using the activation function. Noted that the number of the layers will not change the fact that it is still a linear regression model. We train this model in the same way as we train our CNN model. Figure 5 shows the comparison of the loss between the CNN model and the linear regression model. Noted that the pressure coefficients range normally in (−3, 3) as they are non-dimensionalized by the mean velocity $\bar{u}$.

**Figure 6:**
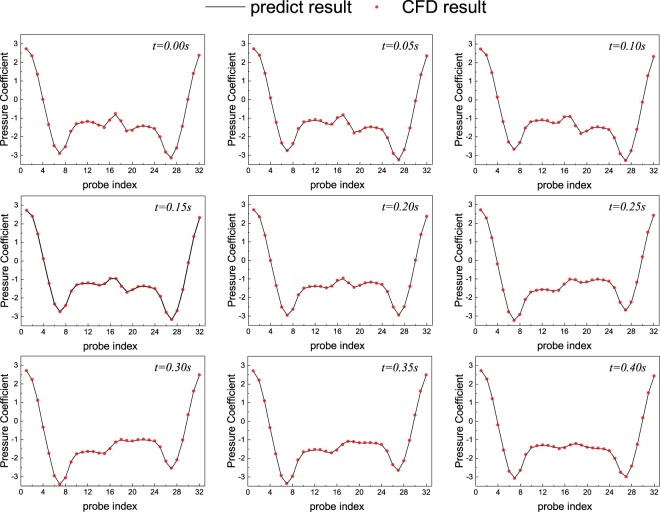
Comparisons of pressure coefficient on 32 points on the cylinder between the model predictions and CFD results with Reynolds number = 10000 at different moments. The red line is regarded as the standard result which we calculated in CFD method before, and the blue line stands for the predicted results through the CNN.

Now we focus on its trained network on the behavior on testing data. When the flow condition changes, the adaptive ability of the network is discussed. The Reynolds number is changed in testing data. By assessing the input of the test data, Table 1 gives the overall RMSE between the predicted pressure coefficient and the CFD-calculated pressure coefficient in CNN model, which is around 3.4 × 10^−3^. The CNN model has better performance on RMSE than the linear regression on both train data and the test data. The CNN performance on Re = 10000 is shown in Fig. 6. As shown in picture, CNN predicted results show good agreements with the CFD results as reference values in the test data set.

**Table 1:** The RMSE on train data and test data.

	Reynolds number	RMSE for CNN	RMSE for linear regression
Train data	1000, 3000, 5000, 7000, 9000	2.5 × 10^−4^	9.0 × 10^−4^
Test data	2000, 4000, 6000, 8000, 10000	3.4 × 10^−3^	1.4 × 10^−2^

### Independence verification of sampling resolution

**Figure 7:**
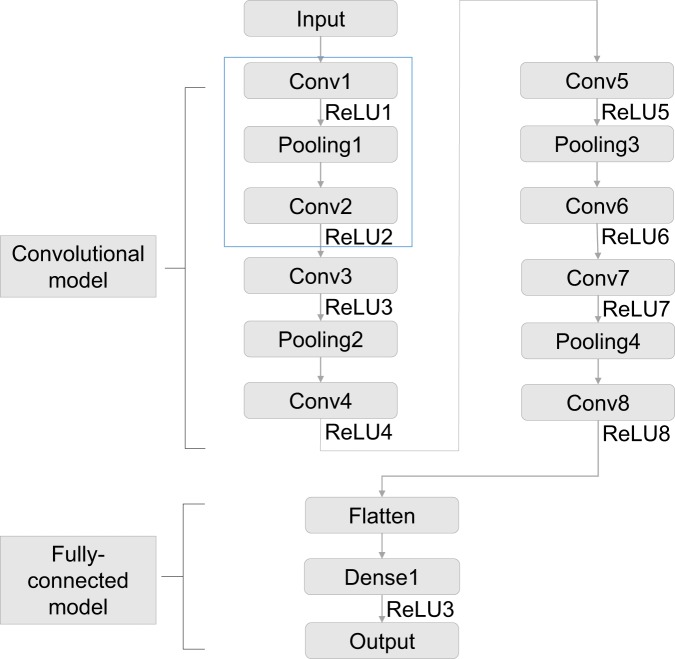
The architecture of the CNN for higher spatial resolution.

In some computational fluid dynamics cases, computing requires to verify the grid independence before further study^17^. Inspired by this CFD computing strategy, similar verification on input data size is carried out with great importance. As for the velocity field chosen as the network input, it is necessary to ensure the size of the velocity information are representative enough to present the flow structure. Therefore, higher spatial resolution of 341 × 101 is interpolated on the same case for the independence study of sampling resolution. To catch up with the larger input size of 341 × 101, more convolutional layers was constructed during this operation. The new CNN configuration with more layers is shown in Fig. 7. The two convolutional layers with one pooling layer is regarded a module, which is repeated three more times to achieve the final architecture.

From the Table 2 the presentation of different spatial resolution can be concluded. When the velocity field changes from 41 × 17 points to 341 × 101 points, the RMSE increases from 2.5 × 10^−4^ to 2.8 × 10^−4^. With the increase of the velocity field points, the error of the network remains small. Though more data is added, the effect on the results of network is negligible. It can be regarded that the verification by refining the spatial resolution of data sampling is achieved while the data of 41 × 17 points satisfies the requirements of calculation. Therefore, considering the cost of calculation time, the network structure with the input size of 41 × 17 is also chosen for further study.

**Table 2:** The presentation on data in different spatial resolution.

	RMSE for training data	RMSE for testing data
41 × 17 points	2.5 × 10^−4^	3.4 × 10^−3^
341 × 101 points	2.8 × 10^−4^	4.2 × 10^−3^

### Discussion on the feature detection mechanism of CNN

#### Interpretation of CNN by visualization

We have mentioned that the flow feature can be extracted from the flow field information, which leads to further derivation to typical parameters as well as main description of the flow field. Now that the above results are the successful attempt to this theory is supposed to be admitted, it suggests that it is meaningful to explore the understanding of convolutional layers for further verification.

Though deep learning is normally regarded as black box for its internal algorithm invisible, convolutional neural network can be visualized. Visualization can help understanding the procedure of CNN training^18^. The visualization technique is to maximize the activation of the filters in different layers, presenting which patterns the data set activates in the convolution process. Therefore, it gives the insight of the features which filters are interested in. To better comprehend the feature detection process, same physical condition data source with higher spatial resolution data is adopted. The architecture of the CNN for higher spatial resolution is described in Section 3.2. Higher spatial resolution points help better describing the physical meaning of the filters and give explanation of the convolutional operation more clearly.

**Figure 8:**
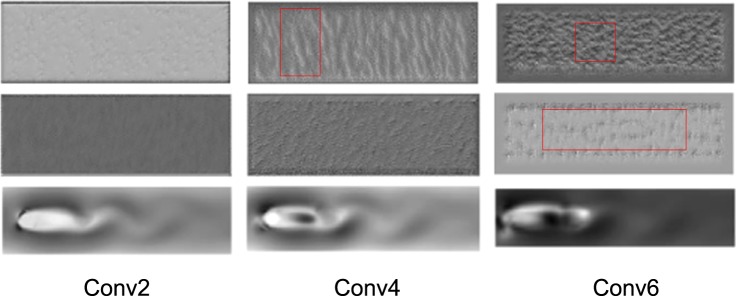
Visualizing of features in a fully trained model on layers: Conv2, Conv4, Conv6.

The first two lines in Fig. 8 shows some of the features in a fully trained model. Visualization on filters emphasizes specific features during the convolutional neural network. When velocity field entered in the input of the network, filters extract corresponding feature and reconstruct to the final output. Six activations of feature visualization separately belonging to 3 layers are shown in Fig. 8. Layer 2 responds to lines. Layer 4 shows variation from layer 2, and is more vortex-like structure. While layer 6 shows the edge of shedding vortex. The last line gives the output of those layer, indicating the contribution of the convolutional layer in a different way. Significant similarities between this CNN in flow field and the common CNN aiming for image recognition is confirmed. Therefore, it is concluded that CNN could detect features in flow field, and apparently is a composite process with gradually complex expression.

#### Regression for reynolds number

Owning the results in Section 3, the successful prediction in pressure coefficient among different Reynolds numbers leads us to further study in the network. Reynolds number is the key dimensionless value label for the flow state. Therefore, the same network configuration is adopted to discuss whether the neural network structure can afford the classification of flow states according to the Reynolds number.

Output of a classification problem is discrete value, which is used to specify which category it belongs to^19^. However, it means the Reynolds number we expect the network to predict should be trained before. Though it is apparently a classification problem, for better understanding of the network we use a regression prospection to deal with it. The network has the same configuration with the previous CNN expect for the output layer, which in this case the output layer only has one neuron indicating the Reynolds number. The regression model is a function that represents the mapping between input variables and output variables. The learning of regression problem is equivalent to function fitting: a function curve is selected to fit the known data well and predict the unknown data well^20^. In this case, this regression will clarify the Reynolds number merely based on the flow velocity, even for those untrained Reynolds number condition.

The training set is the flow around cylinder cases at Reynolds number equals to 1000, 3000, 5000, 7000, and 9000, while the testing set is the flow around cylinder at Reynolds number equals to 2000, 4000, 6000, 8000, and 10000. For each Reynolds number, 300 sets of data are used for training and testing, which is exactly the same as the pressure coefficient prediction condition. Compared with the pressure coefficient prediction case, the change exists in the output of the network from pressure coefficient to Reynolds number.

**Figure 9:**
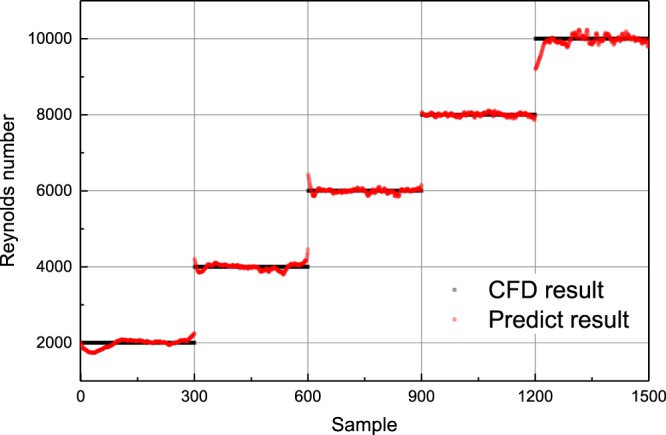
Comparisons of Reynolds number between the model predictions and CFD results for different data set under various Reynolds numbers.

After 100 epochs of training, the RMSE on training data is 2.1, while the testing score reaches to 8.6. Based on the Reynolds number normalization, the RMSE on both training data and testing data are decreased by 4 orders of magnitude, which means the normalized RMSE on training data and testing data is 2.1 × 10^−4^ and 8.6 × 10^−4^, respectively. The predicted Reynolds numbers are nearly identical to the CFD results (Fig. 9), indicating that the network can make good predictions on Reynolds numbers. The result shows that the neural network structure can also figure out the Reynolds number as the main property of the flow. Combined with the pressure coefficient discussed before, we can agree on the flow features learned from the flow velocity field are with enough representativeness. The detected feature in the network could export both flow characteristics and flow property in flow field.

#### Result on transfer learning for high efficiency

The traditional supervised learning meets difficulties when insufficient labeled data for the task is available. In that case, we train the network with the source data and then transfer some layers to the target network^21^. The feature-representation-transfer approach is one of the examples. Owning the labeled data in the source domain, the feature representation can be built through the supervised learning by convolutional neural network. The key for transfer learning is to construct the good feature as well as share the parameters distributions with the target tasks. The transfer learning approach we adopted here is fine-tuning, which is achieved by freezing some of the layers while training the rest layers of the target network. The rest of the weights in the neural network adjust under the target training data set to make sure that this network can achieve better performance in the target domain.

We now focus on how transfer learning improves the computational efficiency in the flow around a cylinder case. The source domain in this transfer learning is the data set in the case of the original parabolic velocity inlet stated in Section 2.1 with Reynolds number ranging from 1000 to 10000. The target domain owns the data set under the condition of parabolic velocity inlet with an additional perturbed term defined as: ${u}_{t}\,=\,u(y)\,\ast \,[1\,+\,\sin \,{\left(\frac{6\pi }{0.41}\times y\right)}^{2}]$. Reynolds numbers in target domain are ranged from 100 to 2000. Admitting the convolutional layer aiming at feature representation, weights in this layer is transferred to the target model. Therefore, weights in those layers from new model are fixed as constant value, leaving the fully connected layer as the only trainable part.

**Figure 10:**
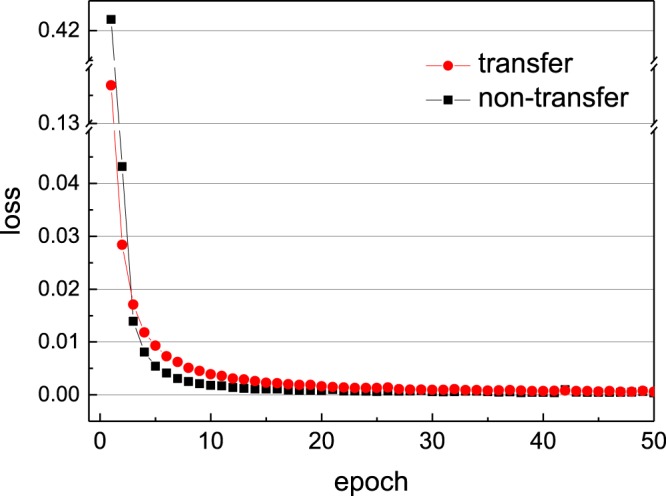
Convergence of the CNN on non-transfer and transfer method: training error for entire test dataset decreases with the increasing number of training epoch.

**Figure 11:**
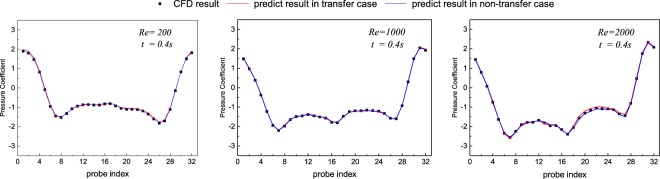
Pressure coefficient on 32 points on the cylinder between the model predictions and CFD results for various Reynolds numbers in transfer case and non-transfer case.

This training approach could reduce the loss more quickly as shown in Fig. 10 and Table 3. The transfer learning method achieves nearly the same accuracy as the traditional one but with significantly lower time cost. Prediction of pressure coefficient on transfer learning approach is shown in Fig. 11. Comparing with the non-transfer learning approach, we can agree on that both methods are able to present the pressure coefficient. Results show that this fixed layers in the convolutional neural network aiming for the feature detection is universal in this kind flow feature recognition problem.

**Table 3:** The presentation on non-transfer and transfer method.

Method	RMSE for train data	RMSE for test data	Time per 50 epoch*
Non-transfer	3.4 × 10^−4^	8 × 10^−4^	3615.7 s
transfer	5.7 × 10^−4^	1.1 × 10^−3^	80.3 s

*This runtime includes the 50 training epoch only. We use an Intel Core i7-8700 CPU at 3.20 GHz, 32.0 GB memory.

## Conclusion

In this work, we propose a convolutional neural network and apply it to the flow analysis. The trained convolutional neural network makes successful prediction on the pressure distributions on the cylinder based on the velocity field in its wake flow. It suggests that the relationship between the flow field and the pressure coefficient in different time steps and Reynolds numbers is deduced. The RMSE of the predicted results and the CFD results is less than 0.004.

This achievement suggests that the CNN could be managed to detect the flow feature. The detected flow feature is correlated with the flow parameters by fully-connected layers. Explanation of how a CNN sees a flow field is preliminarily discussed, as the function of the convolutional layers is visualized. Meanwhile, the regression of the Reynolds number using the same network structure confirmed the CNN could learn the essence of the flow feature.

Further, the generalization ability of the network is investigated by setting up a transfer learning approach of prediction for different velocity inlets and Reynolds numbers, which makes the higher efficiency on the network in both training and testing data sets. With 3 orders of magnitude speedup compared to the non-transfer method, the transfer learning nearly reaches the same accuracy.

In addition, the method we proposed is capable of providing a favorable prediction on the pressure coefficient under different conditions. An obvious next step is to extend the convolutional neural network method to bigger data set of more complex flow fields. The present method and the network system could be an effective strategy for engineering situation in fluid mechanics.
